# Cholera in the Middlesex Hospital

**Published:** 1855-01

**Authors:** 


*Cholera in the Middlesex Hospital.*—It is difficult, without appearing to exaggerate, to convey any adequate idea of the state of the wards during the first four days of September, or of the feelings of admiration with which the House Committee and Medical Officers viewed the noble conduct of all those resident in the establishment. While daylight lasted, the sunshine, though it revealed every minute detail, relieved, by its cheerfulness, some of the horrors of the sad and harrowing sight. But, as night closed in, the dim light shed by a solitary burner, and by the pale moonbeams that struggled through the windows, lent a still more ghastly hue to the livid features, the skinny hands, and the deeply-sunk eyes—in general nearly closed, as if in deaih, but sometimes bloodshot and glaring—of the poor patients, who were screaming in agony or groaning in mortal weakness, on every hand. Add to all this, the sobs and shrieks of new-made orphans and widows, and the clank of the shell, as in its ceaseless round it "vexed the drowsy ear of night"—and you have a very feeble representation of a scene before which many a stout heart might have quailed, and by a single glance at which not a few who presented themselves to be engaged as assistant-nurses, were scared away, without so much as entering the wards. Yet hour after hour, and night succeeding day, did all the members of the Hospital staff—Apothecaries and House-Surgeons, Matron and House-Steward, the few pupils who were in town during the College vacation, sisters, nurses, and porters—discharge, without for a moment shrinking from, tasks the most laborious and the most revolting.—*Edinburgh Monthly Journal of Medical Science, Nov.* 1854.

